# Chionanthus Virginica

**Published:** 1896-06

**Authors:** 


### CHIONANTHUS VIRGINICA:

Eructations, nausea, and vomiting.

Bitter eructations.

Never before felt so sick at the stomach.

Eructations so bitter and sour that I had to hold my mouth open to permit their escape.

Hot, bitter, sour eructations, setting teeth on edge.

Great nausea and retching with desire for stool.

Very violent attack of nausea and a great deal of retching before he could vomit.

Vomitings of very dark-green, ropy, and exceedingly bitter bile, with a single gush.

Vomiting, followed by cold sweat, standing in beads on the forehead, and by extreme weakness.

Sore, weak, bruised feeling all over small of back.

Sore and weak in sacral and lumbar regions; could scarcely walk.

Sore, aching, tired feeling in lower limbs.

Sore, aching and bruised feeling all over body.

Head feels sore and bruised; bruised feeling seems to go deep into the brain.

It is a matter of great regret this remedy has not been fully proved before this time.

In disorders of the stomach and bowels, where the liver is likewise involved, it has but few rivals in the materia medica.

While I do not believe in specifics, yet in jaundice I have cured nine-tenths of my cases with this remedy alone. Improvement is immediate, and I have never seen a case of uncomplicated jaundice fail to be cured by it in a very brief time.

